# The Role of Immunogenetics in the Host–Parasite Interaction of Chagas Disease: Implications for Personalized Medicine

**DOI:** 10.3390/tropicalmed11010002

**Published:** 2025-12-19

**Authors:** Muhammad Hassnain, Syeda Mahnoor Bukhari, Tahira Bibi, Syeda Fakhra Waheed, Monica C. Botelho, Waqas Ahmad

**Affiliations:** 1 University of Veterinary and Animal Sciences, Lahore, Sub-Campus, Narowal 51600, Pakistan; mhassnainmhassnain166@gmail.com; 2 Department of Chemistry, The Women University, Multan 06009, Pakistan; bukharimahnoor86@gmail.com; 3 Department of Botany, Sardar Bahadur Khan Women’s University, Quetta 87300, Pakistan; tahira_botany@yahoo.com; 4 Department of Veterinary Science, Faculty of Veterinary and Animal Sciences, Azad Jammu and Kashmir University of Bhimber, Bhimber 10040, Pakistan; fakhra@ajkuob.edu.pk; 5 Instituto de Investigação e Inovação em Saúde, Universidade do Porto, 4200-135 Porto, Portugal; 6 Department of Health Promotion and Chronic Diseases, INSA—National Institute of Health Dr. Ricardo Jorge, 4000-055 Porto, Portugal; 7 Department of Clinical Sciences, University of Veterinary and Animal Sciences, Lahore, Sub-Campus, Narowal 51600, Pakistan

**Keywords:** Chagas disease, *T. cruzi*, immunogenetics, host–parasite interaction, personalized medicine, immune response, genetic polymorphisms

## Abstract

Chagas disease, caused by the protozoan parasite *Trypanosoma cruzi*, continues to be a significant global health issue, especially in Latin America, with increasing international prevalence due to migration. Despite advancements in diagnosis and treatment, it remains a neglected tropical disease characterized by significant morbidity and mortality, mainly influenced by the complex interaction between parasite diversity and host immune responses. Importantly, the remarkable genetic diversity of *T. cruzi* lineages also contributes to clinical heterogeneity, influencing immune evasion, therapeutic responses, and vaccine feasibility. This review analyzes the impact of immunogenetics on host–parasite interactions in Chagas disease and explores its implications for personalized therapy approaches. Recent research, particularly over the last decade, has indicated that processes including antigenic variation, extracellular vesicle-mediated regulation, and disruption of host signaling pathways facilitate parasite persistence. Host genetic variables significantly influence susceptibility, disease development, and treatment outcomes, including changes in Human Leukocyte Antigen (HLA) genes, cytokine gene polymorphisms, and immunogenetic determinants of cardiac pathology. These findings underscore the potential of immunogenetic markers as tools for prognosis and as targets for personalized therapies. However, there are still considerable research deficiencies. Inadequate comprehension of gene–environment interactions, lack of representation of varied populations, and inconsistencies in study design limit the use of immunogenetic findings in therapeutic settings. At present, the concept of personalized medicine in Chagas disease remains largely aspirational, better understood as a framework for precision public health or stratified interventions guided by host immunogenetic and parasite lineage data. Addressing these issues necessitates comprehensive genomic research, mechanistic investigations of host–parasite interactions, and clinical validation of genetic markers. This study emphasizes the necessity of incorporating immunogenetics into personalized patient management strategies based on existing evidence. This integration has the potential to improve diagnosis, enhance treatment efficacy, and inform preventive interventions, thereby advancing personalized therapy for Chagas disease.

## 1. Introduction

Chagas disease is a major neglected tropical disease caused by the parasite *T. cruzi* [1], affecting approximately 6–8 million people worldwide, mainly in Latin America [2] but increasingly recognized as a global health challenge due to migration [3]. The currently available drugs, such as benznidazole and nifurtimox, show limited efficacy in chronic stages and are frequently associated with adverse reactions [4]. Immunogenetic factors are now recognized as pivotal determinants of disease susceptibility, host–parasite interactions, and disease outcomes, with cytokine and chemokine receptor polymorphisms linked to heterogeneity in immune response, disease severity, and drug toxicity [5,6,7]. Similarly, emerging genomic and transcriptomic evidence highlights that *T. cruzi* employs complex immune evasion strategies, including antigenic variation, sialylation of surfaces, and modulation of host signaling pathways, to ensure persistence [8,9,10]. Despite advances in molecular parasitology, immunology, and pharmacogenomics, translating these findings into a vaccine or stratified therapies remains difficult [11]. The inability to predict aggressive clinical phenotypes, lack of reliable biomarkers and persistent treatment failure rates underscore the need for a deeper understanding of immunogenetic drivers of disease progression [12].

While some genetic polymorphisms have been linked to disease outcomes, the existing evidence is sparse and often population-specific. For example, Batista et al. [13] demonstrated that C–C chemokine receptor type 5 (CCR5) and C–C motif chemokine ligand 5 (CCL5) polymorphisms modulate the clinical course of Chagas cardiomyopathy, whereas Rajendranath Ramasawmy et al. [14] identified Tumor necrosis factor (TNF) and Cytotoxic T-lymphocyte-associated protein 4 (CTLA-4) variants associated with severe cardiac phenotypes. These genes influence leukocyte recruitment, cytokine balance, and T-cell regulatory pathways, which explain their relationship with divergent clinical outcomes. However, many associations failed to replicate across different populations, raising concerns about reproducibility. Epigenetic studies further reveal distinct Deoxyribonucleic Acid (DNA) methylation patterns in blood and cardiac tissues, notably involving Runt-related transcription factor 3 (RUNX3) and T-box transcription factor 21 (TBX21) genes that correlate with disease severity [15,16]. Because RUNX3 and TBX21 govern Th1-driven inflammatory programming, their epigenetic modification may contribute to the heightened immune activation observed in severe cardiomyopathy, although their causal roles remain unclear. Likewise, Franco et al. [17] observed that pharmacogenomic profiles influence benznidazole tolerance, yet no clinical model predicts individual drug response. These inconsistencies reflect broader knowledge gaps where diverse findings remain fragmented and rarely integrated into actionable frameworks [12]. Given the heterogeneous trajectories of Chagas disease, uniform treatment approaches are increasingly inadequate. The failure to synthesize genetic, epigenetic, and pharmacological evidence into cohesive models represents a critical barrier to biomarker discovery, vaccine design and precision-based therapies. Bridging these datasets into a unified narrative is essential to improve patient outcomes and advance translational research.

This review systematically evaluates the role of immunogenetics in host–parasite interactions during Chagas disease, emphasizing implications for personalized medicine. Building on foundational studies such as Machado et al. [18], which demonstrated the interplay between host immune polymorphisms and parasite lineages, and Sánchez et al. [19], who linked Toll-like receptor 4 (TLR4) polymorphisms with vaccine responsiveness, this synthesis adopts an integrative and cross-disciplinary perspective. By combining insights from genetic, epigenetic, and pharmacogenomic research, it delineates how host variability informs stratified therapeutic strategies. Unlike earlier reviews focused narrowly on either host immune pathways or parasite evasion mechanisms, this work emphasizes convergence points where immunogenetic variation shapes both clinical manifestations and treatment outcomes. Furthermore, incorporating recent advances such as genome-to-vaccine approaches [20] and biomarker-driven therapies targeting Transforming growth factor beta (TGF-β) or Poly(ADP-ribose) polymerase 1 (PARP1) pathways [21], this review extends the discussion toward translational precision medicine applications. Ultimately, the goal is to move beyond cataloging genetic associations towards an operational framework that bridges molecular discovery with clinical utility, advancing Chagas disease management from uniform to stratified, and ultimately personalized interventions.

## 2. Immunogenetics and Chagas Disease: A Historical and Current Perspective

### 2.1. Early Understanding of Host–Parasite Interactions in Chagas Disease

**Figure 1:**
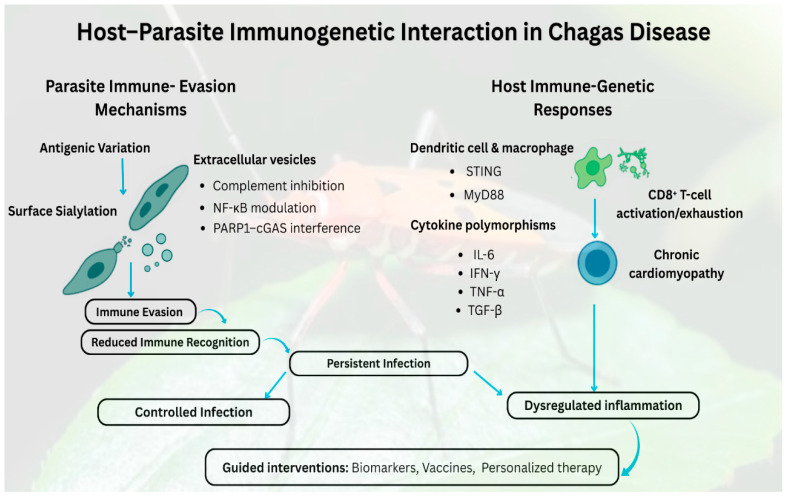
This figure depicts the reciprocal interaction between *Trypanosoma cruzi*’s immune evasion mechanisms and the host’s immunogenetic responses, which together influence the clinical progression of Chagas disease. Parasite strategies, including antigenic variation, surface sialylation, and the secretion of extracellular vesicles that inhibit complement, modulate NF-κB, and interfere with PARP1–cGAS, diminish immune recognition and promote chronic infection. Concurrently, host genetic diversity in innate immune signaling molecules (STING, MyD88) and cytokine genes (IL-6, IFN-γ, TNF-α, TGF-β) affects the activation of dendritic cells and macrophages, influencing CD8^+^ T-cell effector or exhaustion phenotypes, and consequently modulating the progression towards controlled infection, chronic inflammation, or cardiomyopathy. The interplay of these elements delineates how immunogenetic variation influences outcomes ranging from regulated to dysregulated infection and highlights the significance of these pathways for the development of diagnostics, vaccines, and personalized therapy approaches.

The exploration of immunogenetics in Chagas disease has evolved substantially since its discovery more than a century ago [22]. First described by Carlos Chagas in 1909 [23], early investigations were largely clinical and descriptive, focusing on manifestations and epidemiology rather than the molecular basis of host immunity [24]. Although early research recognized the importance of immune responses, the underlying genetic determinants of susceptibility and disease course remain unexplored. The introduction of molecular biology in the late 20th century revolutionized Chagas disease research, allowing scientists to examine the genetic architecture of host–parasite interactions [25]. This transition enables the identification of genetic variations that influence immune competence, susceptibility, and progression, marking a conceptual shift from clinical observation to mechanistic immunogenetic inquiry [26]. This conceptual shift is visualized in Figure 1, which illustrates the dynamic balance between parasite immune evasion strategies and host-cognate immune genetic responses that ultimately dictate infection control, disease progression, and the potential for intervention based on biomarkers. Early evidence of familial clustering of seronegativity and variable clinical outcomes further underscored the need for advanced analytical methods to dissect genetic influences on infection outcomes [27].

Molecular methods such as Polymerase Chain Reaction (PCR) have improved diagnostic accuracy and enabled studies of genetic associations [28,29]. Accurate identification of *T. cruzi* DNA not only enhances diagnosis but also helps investigate the genetic factors associated with disease vulnerability and chronic-phase development. Genes controlling cytokine production are major immunological modulators associated with the pathogenicity of chronic Chagas cardiomyopathy [30]. Because cytokine-regulatory genes shape inflammatory intensity and immune cell activation, their variation provides a mechanistic explanation for the differentiation progression and cardiac involvement observed in chronic Chagas Disease. Subsequent genome-wide association studies (GWAS) have revealed new biological pathways and potential therapeutic targets, thereby strengthening the link between immunogenetic profiling and opportunities in personalized medicine [31]. Nonetheless, in resource-poor settings, standardization and access to molecular diagnostics remain major challenges, limiting their large-scale application for surveillance and clinical management [32,33].

### 2.2. Evolution of Immunogenetics in Chagas Disease Research

Immunogenetics in Chagas disease has evolved from descriptive immunology to a mechanistic, genetics-centric field. Initial studies largely focused on the innate and adaptive immune responses against *T. cruzi*, involving macrophages and T lymphocytes (T cells) or B lymphocytes (B cells) in controlling infection [34,35]. These studies elucidated the mechanisms of immune recognition and elimination of the parasite but rarely investigated the genetic determinants underlying interindividual variation in immune responses [36].

As research progressed, attention shifted towards identifying genetic polymorphisms that influence the strength and type of immune responses. Studies have identified specific host gene polymorphisms that influence cytokine production, antigen presentation, and immune regulatory equilibrium, thereby modulating susceptibility or resistance to infection [37,38,39]. These findings demonstrate that the host’s genetics significantly influences the disease course and clinical diversity [40]. Therefore, combining genetic analysis with immunological approaches is necessary to understand the intricate interplay between host immunity and pathogen persistence [41,42,43].

**Figure 2:**
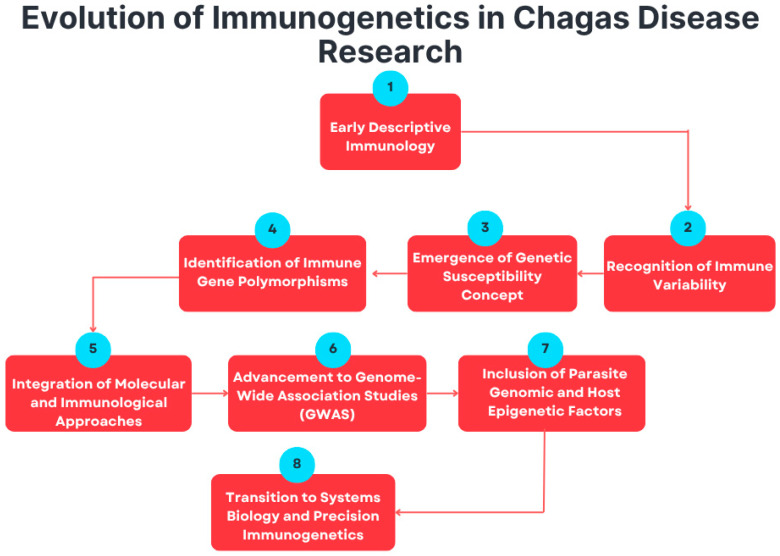
This flowchart summarizes the conceptual progression of immunogenetics in *T. cruzi* research from early descriptive immunology to advanced, system-level precision approaches.

This progressive evolution of immunogenetic research—from descriptive immunology to molecular genetics and precision approaches—is summarized in Figure 2. The emergence of high-throughput genomic and epigenomic technologies has changed the paradigm in this field. The availability of whole-genome and transcriptome sequencing methods now facilitates the systematic identification of the variants driving differences in susceptibility and resistance [15,44,45]. This view of infection in the context of host and parasite genetic diversity goes beyond single-gene associations to a genome-wide perspective, in which both host and parasite genetic diversity together determine disease outcome [46]. Epigenetic modifications, such as DNA methylation and histone modifications, provide an additional layer through which environmental exposure and immune signaling can be linked to gene expression regulation [47].

### 2.3. Recent Immunogenetic Advances

The last five years have seen significant advances in characterizing the immunogenetic basis of Chagas disease. Studies have examined cytokine and chemokine receptor polymorphisms, which mediate host immune signaling [34]. Variants in the CCR5 and C–C chemokine receptor type 2 (CCR2) genes, which are central regulators of leukocyte trafficking, are consistently associated with variable disease expression [48]. For example, CCR5 alleles provide partial protection against chronic Chagas cardiomyopathy (CCC), and specific CCR2 variants modulate chemokine-mediated signaling and the intensity of inflammation, underscoring the role of chemokine receptor variants in controlling infections [13,49].

Epigenetic regulation has emerged as a crucial mode of host–parasite interplay. The DNA methylation profile in primary human diseases provides disease-specific signatures that regulate the expression of immune-related genes, with a focus on cytokine production and T-cell differentiation [15,16]. Transcriptional repression can be induced by hypermethylation of Cytosine–phosphate–Guanine dinucleotide (CpG)-rich regions in promoters, or methylation may promote pro-inflammatory cascades. The modification has also been identified in the RUNX3, TBX21, and Transforming Growth Factor beta 1 (TGF-β1) pathways, associating the methylation status with disease susceptibility and severity [39,49]. Because these genes regulate Th1 differentiation, cytokine balance, and pro-fibrotic signaling, their methylation-driven dysregulation provides a mechanistic basis for the heightened inflammatory and cardiac pathology observed in severe disease. Novel immunogenetic findings are currently being translated into personalized therapeutic approaches. Clinicians can now stratify patients according to susceptibility and resistance, as defined by molecular markers of risk, and modulate therapy accordingly [11,34,50].

## 3. Host–Parasite Interaction: Immune Evasion and Response Mechanisms

### 3.1. Immune Evasion Strategies of T. cruzi

The protozoan parasite *T. cruzi*, which causes Chagas disease, employs numerous sophisticated strategies to evade the host immune response, enabling it to persist within the host for prolonged periods despite robust immune activation [43]. Elucidating these mechanisms is crucial for the development of successful immunomodulatory or vaccine-driven therapies. One of the major mechanisms *T. cruzi* uses to evade immunity is antigenic variation, in which it exhibits a constantly changing surface glycoprotein repertoire [51]. This “antigenic shift” results in non-neutralizing antibody responses that are unable to induce parasite clearance, thereby promoting its expansion during the early phase of infection [52,53]. Another major strategy is surface sialylation, a process in which the parasite transfers sialic acid residues to its glycoproteins via trans-sialidase enzymes [54]. This modification limits the activation and cytotoxicity of Cluster of Differentiation 8-positive T lymphocytes (CD8^+^ T) cells while hiding the parasite from the immune system, thereby facilitating intracellular survival [36].

Extracellular vesicles (EVs) released by *T. cruzi* and infected host cells shape immune responses. These vesicles inhibit complement-mediated lysis and bind Toll-like receptor 2 on host cells, leading to the expression of pro-inflammatory cytokines, including Tumor Necrosis Factor-alpha (TNF-α), Interleukin-6 (IL-6), and Interleukin-1 beta (IL-1β) [55]. Although this reversible activation is beneficial for parasite entry, it contributes to chronic inflammation and tissue damage. Recent studies have identified the PARP1–cyclic GMP–AMP Synthase (cGAS)–Nuclear Factor kappa-light-chain-enhancer of activated B cells (NF-κB) axis as an important pathway linking EV-induced signaling to the persistent activation of macrophages. Activation of this pathway sustains inflammation and fibrotic destruction. In contrast, pharmacological inhibition results in a marked reduction in cytokine release and tissue damage, indicating the therapeutic benefit of PARP1 or cGAS blockade in chronic Chagas disease [56]. Immune evasion strategies can be targeted for new therapeutic options. PARP1 inhibitors can reduce fibrosis by decreasing profibrotic cytokines and metalloproteinases (driving extracellular matrix degradation and remodeling). Their suppression contributes directly to limiting cardiac fibrosis in chronic Chagas disease, while engineered EVs are proposed as vaccine vectors or targeted drug-delivery vehicles that modulate immune cell responses [57].

### 3.2. Host Immune Response to T. cruzi Infection

The immune response to *T. cruzi*, the causative agent of Chagas’ disease, involves a coordinated interplay between innate and adaptive immunity [58]. Dendritic cells and macrophages are crucial for these responses, acting as antigen-presenting cells that coordinate inflammation and parasite suppression. The Stimulator of Interferon Genes (STING) capacity and importance also exist in the innate pathways of activating macrophages and dendritic cells to induce the expression of some major pro-inflammatory cytokines (IL-6, Interleukin-12 (IL-12), and interferon-β [IFN-β]), which mediate inflammation [59].

Moreover, the infection-secreted extracellular vesicles (EVs), which stimulated PARP1–cGAS–NF-κB signaling axis, further provoked macrophage response of IL-6, IL-1β, and TNF-α release, indicating positive amplification for pro-inflammatory environment. Although this response also helps in parasite clearance, its sustained induction leads to chronicity and tissue damage. In addition, cardiomyocytes contribute to the recognition of pathogens via Myeloid differentiation primary response 88 (MyD88)-dependent signaling, leading to CCL5, interferon-gamma (IFN-γ), and TNF-α expression that fuels local immune shielding while also favoring myocardial inflammation [60]. Because MyD88 signaling amplifies pro-inflammatory cascades within cardiac tissue, its activation in cardiomyocytes directly contributes to the transition from acute immune control to chronic inflammatory damage. In the chronic phase of infection, this cytokine-driven inflammation leads to fibrosis and structural disruption, which is typical of chronic Chagas cardiomyopathy.

Adaptive immunity is essential for controlling *T. cruzi*, and CD8^+^ T cells play a central effector role. These cytotoxic T cells kill infected host cells but frequently become functionally exhausted during persistent infection, characterized by decreased proliferation and increased expression of inhibitory receptors [61]. Indeed, antiparasitic treatment in asymptomatic subjects appears to restore CD8^+^ T-cell function partially, confirming the concept of immune rejuvenation [62]. An optimal T helper 1 cell (Th1)/T helper 2 cell (Th2) response is also important for protection. STING-mediated Th1 polarization is characterized by increased levels of IFN-γ and IL-12 and subsequent CD8^+^ T-cell activation [59]. Furthermore, IL-18R/MyD88 signaling favors the generation of cytotoxic CD4^+^ T cells (CD4CTL), mainly in heart tissues, where their frequency positively correlates with the severity of Cardiomyopathy (CM) [63]. Together, these adaptive pathways show that while strong Th1-driven cytotoxic responses are required for parasite control, their sustained activation in cardiac tissue also promotes inflammatory injury, connecting adaptive immunity directly to the progression of chronic cardiomyopathy.

Ongoing Th1-driven inflammatory responses, oxidant stress, and fibrotic remodeling contribute to chronic cardiac disease. Novel nano-immunotherapy targeted to specific antigens has demonstrated potential to reduce inflammation and parasite load and to improve cardiac function [64]. Comparative transcriptomic studies have also shown that, compared with the asymptomatic group, cardiomyocytes from chronic Chagas cardiomyopathy patients display increased expression of pro-inflammatory and oxidative stress-related genes, which might account for altered cardiac injury [65]. Likewise, hepatic immune cells can phenotypically present as pro- or anti-inflammatory early during acute infection because of a complex systemic regulatory environment that affects disease outcomes [66]. Taken together, these results demonstrate that systemic and tissue-specific immune dysregulation, from persistent Th1 activation and oxidative injury in cardiomyocytes to early liver immune polarization, collaborates in the transition from controlled infection toward chronic cardiomyopathic disease.

### 3.3. Immunogenetic Factors Influencing Immune Responses

**Figure 3:**
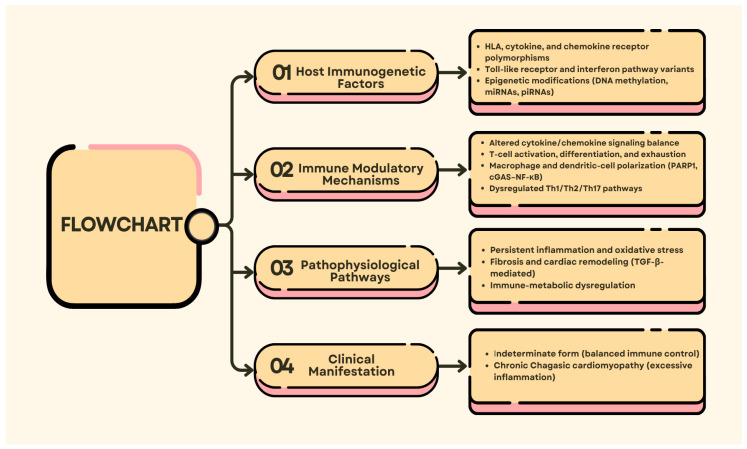
The diagram illustrates the critical host genetic and epigenetic mechanisms underlying immune modulation and the development of *T. cruzi* infection. Immunogenetic factors (cytokine, chemokine, and receptor polymorphisms; DNA methylation; and non-coding Ribonucleic Acid (RNAs)) modify signaling cascades, T-cell activation, and macrophage polarization, with variable inflammatory and fibrotic responses that determine the manifestation of indeterminate or chronic cardiomyopathic forms.

Immunogenetic variation is a major contributor to the host immune response to *Trypanosoma cruzi* infection, affecting susceptibility, disease progression, and clinical course (Figure 3) [41]. Polymorphisms in immune-modulating genes can alter cytokine signaling, receptor sensitivity, and the consequent inflammatory cascades, all of which contribute to the host’s ability to control infection. For instance, mutations in the Interferon-gamma Receptor 1 (IFN-γR1) gene, which encodes the interferon-γ receptor, have been associated with severe clinical phenotypes, and polymorphisms in the Transforming Growth Factor Beta Receptor 2 (TGFBR2) and Interleukin-22 Receptor Subunit Alpha-2 (IL-22RA2) receptors modulate cytokine signaling, altering immune homeostasis and cardiac pathology [18,67]. These polymorphisms result in distinct patterns of cytokine expression and account for some of the interindividual variation in immune response and disease severity. These variants highlight that dysregulation at distinct cytokine-receptor nexus points can converge to alter inflammatory tone and tissue damage, thereby explaining the heterogeneous clinical presentation observed in *T. cruzi* infection.

Interactions between the host and the environment further modify immunogenetic effects. Environmental factors, including reinfection rates, co-infections, nutrition, and vector exposure, may epigenetically alter gene expression for good or ill, thereby ameliorating genetic predispositions [68,69,70]. Conversely, those with pro-inflammatory genetics may experience excessive immune stimulation in response to recurrent infections or inflammatory triggers. Elucidating these gene–environment dynamics is essential for designing stratified prevention and treatment paradigms that incorporate both ecological context and genetic architecture.

The genetic diversity of parasites adds an extra layer of complexity to host immunogenetic associations. *T. cruzi* lineages differ in their virulence factors and surface antigens, such as glycophosphatidylinositol (GPI) anchors and mucins, which can influence host recognition by the immune system and cytokine production [52,54,71]. These strain-restricted molecular signatures shape immune pathway activation and inflammatory potency, highlighting the importance of host genetics and parasite heterogeneity in Chagas disease pathogenesis.

Host genetic polymorphisms heavily influence the clinical phenotype of Chagas disease; however, host factors alone are insufficient to account for infection outcomes. Recent studies have shown that the broad genetic diversity of *T. cruzi* has a major impact on immune kinetics, disease outcome, and response to chemotherapy. Silvestrini et al. [37] demonstrated that parasite lineages account for differences in host immune activation, clinical phenotypes, and treatment susceptibilities, providing evidence that parasite heterogeneity underlies disease complexity. Desale et al. [72] also reported that different *T. cruzi* clones elicited opposite transcriptomic responses in macaque peripheral blood mononuclear cells (PBMCs), leading to strain-specific immune training that drove infection towards control or chronicity.

### 3.4. Parasite Genetic Diversity as a Determinant of Host–Parasite Interaction

**Figure 4:**
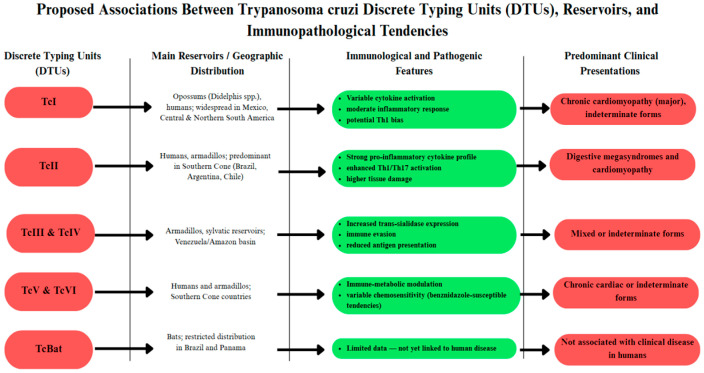
The diagram synthesizes the current understanding of genetic diversity in *T. cruzi* (TcI–TcVI and TcBat), focusing on shared reservoirs, reported immune-modulatory profiles, and preferred, though not exclusive, clinical presentations. The interactions shown are examples of global trends and do not represent absolute immunologic pathways, reflecting the multigenic basis of Chagas disease pathogenesis.

The genetic diversity of *T. cruzi* is a key factor in disease phenotype, immune response, and chemotherapy. Zingales et al. [73] divided *T. cruzi* into seven DTUs (*Trypanosoma cruzi* Discrete Typing Units I–VI (TcI–TcVI) and *T. cruzi* Bat-associated Discrete Typing Unit (TcBat)) (Figure 4), with each Discrete Typing Unit (DTU) being associated with specific ecological habitats, mammalian reservoirs, and clinical outcomes. *T. cruzi* DTU I (TcI) has the widest distribution across Mexico, Central America, and northern South America. In contrast, *T. cruzi* DTU II (TcII), *T. cruzi* DTU V (TcV), and *T. cruzi* DTU VI (TcVI) predominate in the Southern Cone, where they are strongly associated with cardiomyopathy and digestive syndromes. In a Brazilian series, TcII was particularly associated with severe cardiac forms (CHF) and cardiodigestive forms [74]. Velásquez-Ortiz et al. [75] demonstrated that TcI and TcII account for most human infections across continent-wide mapping in the Americas. These lineage-biased distributions and clinical phenotypes indicate that the parasite’s genetic background exerts large effects on host–pathogen interactions and disease diversity.

Mechanistic studies provide insights into how parasite diversity shapes host immunity and treatment outcomes. Barbosa et al. [76] showed that Mexican TcI strains (Ninoa and INC5) modify dendritic cell signaling. In contrast, the Brazilian CL-Brener (TcVI) lineage does not, inducing distinct cytokine profiles and decreased antigen presentation, favoring immune escape. Fonseca et al. [54] also reported that mucin and trans-sialidase expression are strain-dependent, with the TcII and TcVI virulent lineages expressing higher levels of trans-sialidase, which is associated with enhanced thymic damage and immunosuppression. These are characteristic features of lineage-specific immunopathology. Chemosensitivity and vaccine design are also influenced by parasite diversity. Ela, Coral-Almeida [67] found that benznidazole was more effective against TcVI strains than against TcI or TcII, suggesting that parasite genotype contributes to therapeutic variability. Dorn et al. [77] also reported substantial haplotype diversity and geographic segregation within TcI, which has implications for the development of a cross-lineage vaccine.

## 4. Genetic Polymorphisms and Disease Progression in Chagas Disease

The influence of host genetic polymorphisms as modifiers of susceptibility and evolution in CCC is crucial, but their impact differs among populations [78]. Cytokine and chemokine receptor gene variants have differential effects on disease risk across ethnic and geographic regions. Juiz et al. [79] demonstrated that CCR5 variants had contradictory effects in Argentinean populations; rs1800024T protected against risk, whereas rs41469351T-associated risk showed different patterns among Wichi and non-Wichi descendants. In addition, Frade et al. [47] conducted an extensive case–control study in the Brazilian population and found that several host genetic variants were associated with CCC. In particular, the *CCR5* rs3176763 C/C genotype was associated with protection, whereas the C–C Motif Chemokine Ligand 2 (*CCL2*) rs2530797 A/A and Toll–Interleukin 1 Receptor (TIR) Domain-Containing Adaptor Protein (*TIRAP*) rs8177376 A/A genotypes were associated with increased susceptibility to CCC. Since these genes control leukocyte trafficking and pro- versus anti-inflammatory signaling, the variable function of these processes provides a mechanism for why some polymorphisms predispose to myocardial injury while others protect. These findings suggest that polymorphisms in genes involved in chemokine signaling and innate immunity are associated with the host immune response to *T. cruzi* infection, potentially influencing the development of cardiomyopathy from the indeterminate form. Batista et al. [13] also reported a protective effect of the *CCL5* rs2107538 polymorphism in CCC in a Brazilian cohort; however, no association was observed in Colombian patients. Functionally and experimentally, their results showed that C–C Chemokine Receptor 1 (*CCR1*)-inducing Interleukin-10 (IL-10)-dominant responses led to myocardial protection, whereas CCR5-releasing TNF-producing cells contributed to inflammatory damage, underscoring the contrasting regulatory activities of *CCR1* versus CCR5 pathways in disease. In contrast, Alvarado-Arnez et al. [80] demonstrated no significant association between Tumor Necrosis Factor Receptor 1 (TNFR1), Tumor Necrosis Factor Receptor 2 (TNFR2), or TNF variants and CCC progression, revealing population-specific heterogeneity in cytokine-related gene effects.

In addition to chemokine networks, single-nucleotide polymorphisms (SNPs) in innate immunity genes also influence disease outcomes. Ramasawmy et al. [81] reported that heterozygosity for the MyD88-Adaptor-Like protein (MAL)/TIRAP S180L polymorphism decreases the risk of CCC by regulating Toll-like receptor signaling and maintaining homeostatic inflammatory balance. Dias et al. [82] reported that the Cytotoxic T-Lymphocyte Associated Protein 4 (CTLA-4) −1722CC genotype and CCA haplotype were significantly associated with the indeterminate clinical form of Chagas disease, suggesting that alleles linked to higher CTLA-4 expression may contribute to a balanced immune regulation and reduced pathology. Another study by Clipman et al. [83] implicated additional inflammasome-related genes (NOD-Like Receptor Family Pyrin Domain-Containing 1 (NLRP1) (rs11651270) and Caspase Recruitment Domain Family Member 11 (CARD11) (rs6953573)) in CCC progression, suggesting inflammasome-driven inflammation as a key mechanism in disease pathogenesis. Together, these polymorphisms alter innate immune signaling thresholds, impacting TLR activation, checkpoint inhibitor activity, and inflammasome assembly, providing a mechanism for the differential association of these polymorphisms with protection, indeterminate disease, or progression to cardiomyopathy.

The clinical translation of these genetic findings has been emphasized in recent studies. Ordóñez et al. [84] established a loop-mediated isothermal amplification (LAMP) method targeting a highly repetitive satellite DNA sequence of *T. cruzi*, which could be used for sensitive early detection in limited-resource areas. Franco et al. [17] identified three SNPs (rs1518601, rs11861761, and rs34091595) on chromosome 16 that were strongly associated with benznidazole-related adverse drug reactions in Chagas disease patients, although the function of the implicated gene (LOC102724084) remains unknown, highlighting the need for further pharmacogenomic characterization. Gómez et al. [85] demonstrated that asymptomatic individuals exhibit signature immunogenetic expression profiles and upregulation of Signal Transducer and Activator of Transcription 1 (STAT1) and interferon type II networks, associated with protection against the disease. These expression patterns could serve as predictive biomarkers and contribute to the development of personalized medicine. Collectively, these diagnostic, pharmacogenomic, and immunogenetic markers demonstrate that the diversity of molecular variation plays a critical role in shaping early detection, drug safety, and host-response profiling, thereby linking genetic discovery to clinically actionable stratification.

## 5. Epigenetic Modifications in Chagas Disease: Regulation of Host Defense Mechanisms

Epigenetic regulation has become a determinant of the pathogenic course and immunomodulation in Chagas disease. DNA methylation-based profiling has revealed concrete epigenetic factors that distinguish mild from severe CCC phenotypes. Brochet et al. [86] identified more than 6700 differentially methylated loci in peripheral blood and a 33-CpG methylation predictor for disease stage with high sensitivity. Pathway enrichment analyses identified ion transport regulation, cytoskeleton remodeling, and Mitogen-Activated Protein Kinase (MAPK) signaling as potential pathways underlying these circulating methylation markers of systemic inflammation. Tissue-level analyses complemented these findings by illustrating the underlying regulatory mechanisms. Brochet et al. [15] identified 1407 transcripts with varying expression levels and 92 methylation-regulatory regions in myocardial tissue, most of which were in the promoters of immune-related genes. Transcription factors, including RUNX3, TBX21, and Early B-cell Factor 1 (EBF1), were epigenetically modulated, potentially associated with immune polarization toward Th1/IFN-γ in myocarditis. The transcription factors themselves direct Th1 lineage commitment and the production of pro-inflammatory cytokines; therefore, their epigenetic dysregulation provides a mechanistic basis for the extreme inflammatory environment observed in severe CCC. Building on these findings, the methylation signatures in blood identified by Brochet et al. [86] indicated that peripheral blood DNA methylation is a highly accurate biomarker for distinguishing asymptomatic from CCC patients and for staging disease severity.

Diverse transcriptional and epigenetic networks intersect to maintain chronic inflammation in CCC patients. Methylation alterations in the Transcription Factor Binding Sites (TFBSs) of RUNX3 and TBX21 induce Th1-skewed immune implication and contribute to sustained cardiac inflammation. Choudhuri et al. [56] showed that the PARP1–cGAS–NF-κB axis intersects with chromatin regulation, despite not being a classic epigenetic pathway, to shape macrophage activation during *T. cruzi* extracellular vesicle release. As this axis potentiates DNA-sensing and NF-κB-dependent inflammatory pathways, its sustained activation can drive macrophage-mediated tissue injury during chronic disease. Wen et al. [87] also demonstrated that hyperactive PARP1 impairs mitochondrial DNA integrity in cardiomyocytes, exacerbating oxidative stress and structural damage; repressing PARP1 has been reported to help restore mitochondrial bioenergetics and limit cardiac injury. At the post-transcriptional level of regulation, a large-scale myocardial microRNA (miRNA)–messenger Ribonucleic Acid (mRNA) profiling study conducted by Laugier et al. [88] identified approximately 70 miRNAs in the CCC myocardium, including “master regulators”, such as miR-125b-5p, miR-15a-5p, miR-296-5p, miR-29c-3p, and miR-103a-3p, which control dozens of genes related to fibrosis, inflammation, hypertrophy, and oxidative stress. These results highlight the pivotal role of miRNAs in an additional layer of epigenetic regulation that promotes IFN-γ-, TNF-, and NF-κB-mediated pathogenic programs in CCC.

Epigenetic mechanisms also offer new therapeutic targets for both the host and the parasite. Ferreira et al. [89] identified *T. cruzi* lysine deacetylase Sirtuin 2 (Class III) as a potential drug target, with the inhibitor CDMS-01 showing stronger in vitro antiparasitic activity, suggesting the possibility of parasite-directed epigenetic drug design. Laugier et al. [90] reported widespread DNA methylation changes in heart-immune genes, including RUNX3, highlighting methylation as a biomarker and a target for host immunomodulation. Furthermore, Cooley et al. [91] demonstrated that PIWI-interacting RNAs (piRNAs) target molecules in the IL-6 signaling pathway during early infection, indicating another level of non-coding RNA in host–parasite interactions. Because piRNAs regulate post-transcriptional gene silencing, their targeting of IL-6-related molecules may moderate early cytokine dynamics and downstream inflammatory responses. In contrast, sirtuin 2 inhibitors have advanced to established drug pipelines, whereas piRNA-guided interventions remain at the experimental stage, thus illustrating the translational divide between mechanistic understanding and intervention.

Further insights into parasite-directed epigenetics have demonstrated that *T. cruzi* undergoes profound chromatin modifications throughout its life cycle. Using Formaldehyde-Assisted Isolation of Regulatory Elements sequencing (FAIRE-seq) and chromatin accessibility profiling, Lima et al. [92] showed that epimastigotes are characterized by open chromatin with an enrichment of activating histone marks such as Histone H3 lysine 4 methylation (H3K4me), and metacyclic forms have globally compacted chromatin marked by repressive marks such as Histone H3 lysine 9 trimethylation (H3K9me3) and Histone H3 lysine 27 trimethylation (H3K27me3). These stage-specific patterns are especially evident at Transfer RNA (tRNA) loci, where developmental differences in chromatin accessibility indicate regulatory control of nearby transcription units and differentiation. Rosón et al. [93] demonstrated that the histone variant Histone H2B variant V (H2B.V) is located in the transcription-related regions of the genome, physically interacts with Bromodomain Factor 2 (BDF2), and is essential for chromatin architecture, parasite differentiation, and host cell invasion—experimental reduction in H2B.V levels promote metacyclic differentiation and infectivity to mammalian cells, attesting that histone variant dynamics are a factor in virulence and life-cycle progression.

## 6. Immunogenetics and Vaccine Development for Chagas Disease

Efforts to develop an effective vaccine against *T. cruzi* are increasingly influenced by direct human and computational genomics-based approaches that tackle both parasite diversity and host variability [94]. Recent translation focuses on prediction modeling, conserved epitope discovery, and regulated immune modulation, which have the potential to augment protective immunity. Becker et al. [95] reported that Trypomastigote Complement 24 kDa Antigen (Tc24), a trypomastigote excretory–secretory antigen, is a conserved immunogen across TcI isolates in Latin America, suggesting it as a target for population-wide vaccine candidates.

In parallel with Tc24, trans-sialidase (TS)-based subunit vaccines have shown that rationally engineered antigens can elicit both robust effector and regulatory responses in humans. Prochetto et al. [96] stated that inoculation with a truncated, catalytically inactive fragment of Trans-sialidase fragment (TSf), adjuvanted in Immune-Stimulating Particle Adjuvant (ISPA), led to pronounced Th1-biased immunity manifesting TS-specific antibodies, as well as delayed-type hypersensitivity and higher IFN-γ production by CD8^+^ T cells. It also modulated regulatory circuits by decreasing the number of myeloid-derived suppressor cells and increasing the frequency of Cluster of Differentiation 4-positive Forkhead box protein P3-positive regulatory T cells (CD4^+^Foxp3^+^ T regulatory cells) upon challenge. This combination provided ~90% protection and markedly reduced parasitemia in mice, compared with ~40% survival in the control group, demonstrating that vaccine antigens can be engineered to combine effector clearance with controlled immunoregulation. Within the same experimental approach, other antigens, such as cruzipain and Glycoprotein 82 (gp82), have also been proposed as promising components or supports for a broader multiantigenic repertoire in multicomponent or combined vaccines against *T. cruzi*.

Computational vaccinology has further developed this concept by using in silico prediction to identify conserved T-cell epitopes across various *T. cruzi* lineages. Michel-Todó et al. [97] developed cocktail multi-epitope vaccines predicted to induce cross-protective CD4^+^ and CD8^+^ T-cell responses with minimal cross-reactivity with host proteins. This “pan-vaccine” approach provides a feasible strategy for addressing the diversity of parasite strains.

Building on these epitope-focusing strategies, several novel platforms are currently being investigated for multi-antigen formulations in clinically relevant scenarios. Mancino et al. [98] employed mRNA-encapsulating lipid nanoparticles containing the mRNAs of Tc24 and the amastigote antigen Amastigote Surface Protein-2 (ASP-2) in a chronic murine model of Chagas disease. They observed reductions in parasite burden and cardiac inflammation with both monovalent and bivalent mRNA vaccines. Tc24- and Tc24/ASP-2-mRNA had a therapeutic effect on infection, underscoring that mRNA is well suited not only for prophylaxis but also for therapeutic vaccination and provides a robust system to rapidly modulate multicomponent *T. cruzi* vaccine candidates.

Additional approaches are being developed for the therapeutic vaccination of established infections. Jones et al. [99] developed a “vaccine–chemotherapy” approach combining controlled immunization with anti-parasite chemotherapy, resulting in balanced Th1/Th2/T helper type 17 (Th17) responses, decreased cardiac inflammation, and enhanced parasite control during chronic disease. This strategy demonstrates that immune modulation can enhance pharmacologic activity and minimize tissue pathology.

These developments are accompanied by an increasing understanding of how innate immunogenetic pathways can be leveraged as targets for adjuvant treatment. Rodrigues et al. [100] demonstrated that TLR2, TLR4, Toll-Like Receptor 7 (TLR7), or Toll-Like Receptor 9 (TLR9) perceive discrete pathogen-associated molecular patterns of *T. cruzi* that cooperatively promote the production of IL-12, TNF-α, and IFN-γ, as well as dendritic cell maturation and CD8^+^T cell priming, thus explaining the use of such adjuvants as TLR4 ligands in Th1-biased adjuvant combinations. More recently, Sanchez Alberti et al. [101] reported that a trivalent chimeric immunogen formulation (Traspain), combined with STING agonist adjuvant Cyclic di-adenosine monophosphate (c-di-AMP), induced a robust Th1/17-skewed response along with enhanced production of IFN-γ, Interleukin-2 (IL-2), and Interleukin-17 (IL-17); multifunctional CD4^+^ T cells; and pathogen-specific CD8^+^ T cells, which resulted in the control of parasitemia and chronic cardiac inflammation. This work demonstrates that activating cytosolic DNA-sensing pathways, including STING, constitutes a rational adjuvant approach based on well-established innate signaling axes previously linked to *T. cruzi* pathogenesis and with potential for next-generation Chagas vaccines.

## 7. Immunogenetic Research and Therapeutic Advancements in Chagas Disease

The chronic phase of Chagas disease is characterized by myocardial inflammation, fibrosis, and, later, cardiac insufficiency [102]. Immunogenetic investigation has now moved to translating molecular insights into therapeutic applications that could modify the course of the disease and benefit cardiac function. In recent studies, Transforming Growth Factor-β (TGF-β) signaling is a central regulator of cardiac remodeling during *Trypanosoma cruzi* infection. Ferreira et al. [21] reviewed that pharmacological inhibition of TGF-β with GW788388 in experimental models reversed myocardial fibrosis and reestablished conduction pathways. Despite being efficacious at reducing extracellular matrix deposition, kinase-mediated inhibition might lead to toxicity issues, as cautioned by Waghabi et al. [103]. Based on these results, Ferreira et al. [49] recently reinforced that several TGF-β pathway inhibitors, such as small-molecule Transforming Growth Factor beta Receptor I (TβRI) antagonists, such as SB-431542, GW788388 and the neutralizing monoclonal antibody 1D11, can lower parasite burden and repair connexin-43 organization, preserve cardiac electrical impulse conduction, and mitigate fibrosis in animal models, while high circulating TGF-β levels in patients correlate with a worse prognosis, making this cytokine an attractive therapeutic target, in addition to being a biomarker of disease progression.

Parallel studies have also discovered novel immunomodulatory and pharmacological combinations for resolving chronic inflammation. Choudhuri and Garg also demonstrated that macrophage activation could be inhibited by blocking the PARP1/Activator Protein 1 (AP-1) axis, thereby preventing fibroblast-driven fibrosis, suggesting that PARP1 inhibition may also play a role as an adjunct therapy. Because the PARP1/AP-1 pathway enhances inflammatory gene expression and fibroblast activation, its inhibition provides a mechanistic rationale for limiting chronic tissue remodeling in Chagas cardiomyopathy. Konduri et al. [104] engineered a dendritic cell vaccine to overexpress Src Homology Region 2 Domain-containing Phosphatase 1 (SHP-1) to promote CD8^+^ IFN-γ responses, reduce parasite burden, and reduce cardiac pathology. Pereira et al. [105] found that treatment with benznidazole in combination with low-dose aspirin activated lipoxin-mediated antifibrotic signaling, preventing cardiovascular dysfunction, indicating a synergy between antiparasitic drugs and immunoregulatory compounds.

In addition to these pathway-specific and combinatorial approaches, host-directed antifibrotic targets have been identified in experimental models. Pineda et al. [106] demonstrated that *T. cruzi*-infected mice lacking galectin-3 showed significantly reduced inflammatory cell infiltration and collagen deposition in the heart, despite similar parasite loads, suggesting galectin-3 as a pivotal molecule in pathological fibrosis and, therefore, an attractive host-directed target to restrict cardiac remodeling during chronic Chagas cardiomyopathy. Not all candidate pathways display strong genetic signals from an immunogenetic perspective; however, in a large Brazilian case–control study by Alvarado-Arnez et al. [80], no substantial effect of multiple cytokine-related polymorphisms (covering Transforming Growth Factor Beta (TGFB), IL10, TNF, TNFR1, and TNFR2) on progression to cardiomyopathy after correction for the large number of tests undertaken was identified, highlighting again that we believe clinical heterogeneity results from complex parasite–host–environmental interactions rather than individual high-impact cytokine variants.

The pathway to clinical translation is now predominantly driven by precision frameworks with biomarker-based strategies. Pinazo et al. [107] emphasized that current therapeutic markers are mainly parasite- or immune-associated rather than host-derived genetic, while Balouz et al. [108] stated that new omics approaches (proteomics, metabolomics, high-throughput epitope mapping) can combine parasite and host-immunogenetics data for personalized treatment decisions. While still investigational and being included in integrated workflows, such integrative pipelines herald the evolving paradigm from empirical therapy to biologically stratified care. Collectively, immunogenetic and therapeutic research is redefining Chagas disease management. Targeted pathway inhibition, multi-drug immunoregulation, and omics-based biomarker validation together lay the groundwork for patient-specific interventions, marking a shift toward precision medicine guided by both host genetics and parasite biology [109].

Recent cardiomyocyte-targeted transcriptomic analyses support this precision paradigm by showing that distinct *Trypanosoma cruzi* lineages leave distinct molecular imprints in the host myocardium. Candray-Medina et al. [110] demonstrated that when HL-1 cardiomyocytes are infected with the TcI/TcII strains, common pathways related to hypertrophy and apoptosis are upregulated. This occurs alongside the activation of MAPK signaling and oxidative stress-related proteins during both stages of parasite development. In contrast, unique regulatory pathways, such as those involved in glutathione metabolism, one-carbon metabolism, and nitrogen compound metabolism, were observed only in infections caused by TcI/TcII. This finding indicates lineage-specific metabolic reprogramming associated with an increased risk of cardiomyopathy. The transition also identified growth/differentiation factor 15 (GDF15) as a key upregulated gene and a putative biomarker of cardiomyocyte stress, suggesting that transcriptomic signatures could nominate progression markers and druggable metabolic or oxidative pathways for genotype-specific host-directed therapies targeting Chagas disease progression. A consolidated synthesis of these findings is presented in Table 1, which summarizes the principal therapeutic strategies, mechanistic pathways, experimental systems, and translational implications reported across preclinical and clinical studies.

## 8. Future Directions: Integration of Immunogenetics into Personalized Medicine for Chagas Disease

**Figure 5:**
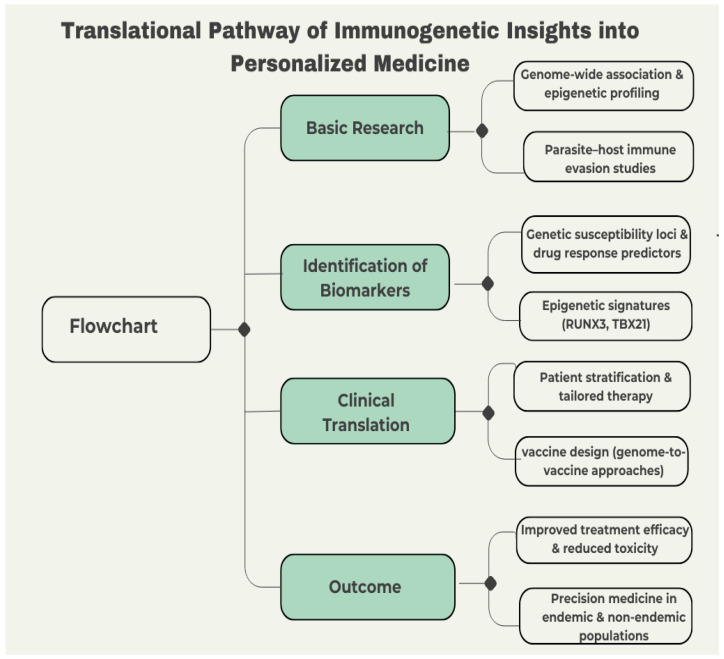
The flowchart illustrates the steps from fundamental research to clinical implementation. This includes early genome-wide association and epigenetic profiling, biomarker development (including susceptibility loci and epigenetic signatures such as RUNX3 and TBX21), patient stratification, vaccine design, and therapeutic effectiveness. The objective is to implement precision medicine in both endemic and non-endemic populations.

Future studies on Chagas disease should evolve from elucidating descriptive immunogenetics to clinically relevant frameworks that incorporate host, parasite, and environmental factors [120]. Though significant progress has been made at the molecular level, there is consensus that the critical issue today is to translate theoretical advances into personalized patient care. Figure 5 illustrates this translational pathway—from basic immunogenetic discoveries and biomarker identification to clinical application and precision medicine outcomes—summarizing how genomic, epigenetic, and immunological insights can be integrated into patient-specific strategies.

One major area is the development of predictive models that integrate host genetic susceptibility with ecological and epidemiological correlations. Case–control studies that integrate vector exposure, reinfection frequency, and comorbidity profiles could lead to risk-stratification systems applicable to endemic populations [121,122,123]. Such multidimensional modeling would allow the identification of those likely to progress to severe cardiomyopathy and guide region-specific surveillance and therapeutic approaches.

Overcoming the barriers to clinical translation will determine the effectiveness of these efforts. Implementation studies are needed to validate candidate biomarkers in genetically and geographically diverse cohorts, with parallel scale-up of laboratory infrastructure and genetic screening capacity for implementation in low-resource settings [124,125]. For these purposes, collaborative consortia that connect institutions from endemic countries to international research networks will be crucial for maintaining data harmonization, training, and equitable access to emerging technologies.

The evolution should progress even more rapidly, given the newer analytic platforms. Artificial intelligence and machine learning models can integrate multi-omics (genomic/transcriptomic/proteomic) and clinical data to predict therapeutic response or disease progression with high accuracy [126,127,128]. These computational pipelines may inform prospective adaptive clinical trials by iteratively improving treatment regimens guided by a patient’s biomarker profile.

However, interpreting immunogenetics in precision medicine raises various ethical and societal issues. Genetic testing in disadvantaged populations may face stigma and unequal access to new interventions without adequate governance [129,130,131]. Therefore, international financial frameworks and culturally appropriate consent processes need to inform the implementation of omics-based screening and data sharing to ensure programs are fair and accountable.

Collectively, these advances herald a future in which immunogenetic risk becomes the cornerstone of a stratified rather than a uniform therapeutic strategy. Using validated biomarkers, computational integration, and ethically informed rollout, it may be possible for Chagas disease management to move away from empirically based treatment toward personalized, evidence-based interventions that increase the likelihood that patients do well while being fair to the endemic regions [132].

## 9. Conclusions

This review synthesizes current knowledge of host–parasite immunogenetic interactions in Chagas disease, emphasizing how genomic variation in both the host and the parasite influences infection outcomes and treatment options. Recent research has demonstrated that *T. cruzi* uses intricate mechanisms to maintain persistence, whereas host immunogenetic diversity significantly influences susceptibility and clinical manifestations. Genetic and epigenetic findings have recently created a framework for the precision-based management of Chagas disease. The integration of genetic, transcriptomic, and immunological data facilitates the identification of potential biomarkers that may inform personalized risk assessment, prognosis, and therapeutic selection. Simultaneously, environmental and socio-epidemiological factors, such as reinfection pressure and ecological exposure, influence these genetic consequences, underscoring the need for gene–environment modeling in future translational research. Despite substantial advancements, significant gaps remain. Most immunogenetic data originate from Latin American cohorts, limiting their global applicability and cross-population validation. Standardized diagnostic techniques, longitudinal follow-up, and the inclusion of underrepresented regions are crucial for obtaining reproducible, causally interpretable data. The roles of epigenetic regulation and non-coding RNA pathways in immunological remodeling are poorly defined and require thorough investigation. The concept of “personalized medicine” for Chagas disease is currently more of an aspirational framework than a clinical reality. Resource limitations restrict therapeutic options, and the lack of confirmed genetic indicators currently hinders personalized treatment. Nonetheless, the framework of stratified or precision medicine provides a scientifically substantiated trajectory for the upcoming decade, connecting fundamental discoveries to individualized patient care via collaborative, ethically driven, and technologically enhanced research. In conclusion, advancing immunogenetic understanding of Chagas disease will improve clinical therapy and strengthen global control measures for this neglected tropical disease. Connecting laboratory findings to practical applications is a crucial step toward realizing the potential of precision medicine in both endemic and developing regions.

## Figures and Tables

**Table 1:** Key Therapeutic Advances in Chagas Disease.

Study	Therapeutic Strategy/Target	Model System	Main Mechanistic Finding	Key Implication
Ferreira et al. [111]	TGF-β neutralization (1D11 monoclonal antibody)	In vitro cardiomyocytes; acute and chronic mouse models	Reduced *T. cruzi* invasion, parasite load, conduction defects, and fibrosis	TGF-β blockade offers dual antiparasitic and antifibrotic benefit
de Freitas Souza et al. [112]	Galectin-3 inhibition (genetic; N-acetyllactosamine (N-Lac))	Chronic mouse model; human hearts; fibroblast assays	Galectin-3 (Gal-3) drives fibroblast proliferation and collagen I; inhibition reduces inflammation and fibrosis	Gal-3 is a key host-directed antifibrotic target
Choudhuri et al. [113]	PARP1/AP-1 pathway inhibition	Infected macrophages/fibroblasts; chronic mouse model	PARP1 promotes Matrix Metalloproteinase (MMP) 2/9-TGF-β axis and myofibroblast differentiation; inhibition reduces fibrosis	PARP1 is a central regulator of profibrotic macrophage signaling
Staneviciute et al. [114]	Benznidazole + rapamycin nanotherapy	Chronic cardiomyopathy mouse model	Combined therapy lowers parasite load, normalizes TNF-α, increases IL-10, improves cardiac function	Nanocarrier-enabled immunomodulation enhances benznidazole efficacy
Carrillo et al. [115]	AT-RvD1 pro-resolving therapy ± low-dose benznidazole	Chronic Wild-Type (WT) and FPR2−/− mice	Aspirin-Triggered Resolvin D1 (AT-RvD1) via Formyl Peptide Receptor 2 (FPR2) reduces inflammation, fibrosis, hypertrophy, and parasite burden	Resolution-phase therapy improves cardiac injury and pairs well with reduced-dose Benznidazole (BZN)
Konduri et al. [104]	SHP-1-silenced dendritic cell vaccine (Tc24/Trypomastigote Surface Antigen-1 (TSA-1))	Acute Bagg Albino C (BALB/c) model	Strong CD8^+^ IFN-γ^+^ responses; 76→99% reduction in parasite burden and pathology	Dendritic Cell (DC)-based vaccines can prevent early cardiac damage
Lannes-Vieira [116]	Multi-target immunomodulation (anti-TNF, Pentoxifylline (PTX), TGF-β blockade, low-dose BZN)	Preclinical mouse models	TNF/TGF-β pathways and TNFR1^+^CD8^+^ T cells drive CCC; PTX and cytokine blockade rebalance inflammation and improve conduction/fibrosis	Combination therapy targeting inflammatory networks is mechanistically justified
Candray-Medina et al. [110]	Omics-guided target discovery	Atrial cardiomyocyte cell line (HL-1) cardiomyocytes infected with TcI/TcII/TcVI	Cardiovirulent strains upregulate Reactive Oxygen Species (ROS), glutathione/nitrogen metabolism; GDF15 strongly induced	Identifies metabolic/oxidative pathways and GDF15 as therapeutic and biomarker targets
Pinazo et al. [117]	Biomarker framework for treatment monitoring	Systematic review of human Chagas Disease studies	No single marker sufficient; combined molecular/serologic/parasitological panels needed	Supports standardized multi-marker endpoints for trials and follow-up
Budni et al. [118]	Carvedilol (antioxidant β-blocker)	Human patients with chronic Chagas cardiomyopathy	Reduced protein carbonyls and lipid peroxidation; improved redox balance via ROS/Reactive Nitrogen Species (RNS) scavenging and modulation of antioxidant enzymes	Oxidative stress attenuation with carvedilol may complement antiparasitic/immunomodulatory therapy in chronic CCC
Angel et al. [119]	Inhibition of *T. cruzi* IMPDH (AVN-944)	Recombinant enzyme assays; infected Rat cardiomyoblast cell line (H9c2) cardiomyoblasts	AVN-944 showed sub-micromolar inhibition of *T. cruzi* Inosine Monophosphate Dehydrogenase (TcIMPDH) and superior antiparasitic activity/selectivity vs. benznidazole	Inosine Monophosphate Dehydrogenase (IMPDH) is a validated metabolic drug target; AVN-944 is a promising candidate for genotype-agnostic antiparasitic therapy

## Data Availability

No new data were created or analyzed in this study.

## Abbreviations

**Acronym**	**Full term**
ALK5	Activin Receptor-Like Kinase 5
AP-1	Activator Protein 1
ASP-2	Amastigote Surface Protein-2
AT-RvD1	Aspirin-Triggered Resolvin D1
B cells	B lymphocytes
BALB/c	Bagg Albino C
BDF2	Bromodomain Factor 2
BZN	Benznidazole
CARD11	Caspase Recruitment Domain Family Member 11
cGAS	cyclic GMP–AMP Synthase
CCC	Chronic Chagas Cardiomyopathy
CCL2	C–C Motif Chemokine Ligand 2
CCL5	C–C Motif Chemokine Ligand 5
CCR1	C–C Chemokine Receptor 1
CCR2	C–C Chemokine Receptor 2
CCR5	C–C Chemokine Receptor 5
CD4^+^ T cells	Cluster of Differentiation 4-positive T cells
CD4CTL	Cytotoxic CD4-positive T cells
CD8^+^ T cells	Cluster of Differentiation 8-positive T cells
c-di-AMP	Cyclic di-adenosine monophosphate
CM	Cardiomyopathy
CpG	Cytosine–phosphate–Guanine dinucleotide
CTLA-4	Cytotoxic T-Lymphocyte-Associated Protein 4
DC	Dendritic Cell
DTU(s)	Discrete Typing Unit(s)
DNA	Deoxyribonucleic Acid
EBF1	Early B-Cell Factor 1
EVs	Extracellular Vesicles
FAIRE-seq	Formaldehyde-Assisted Isolation of Regulatory Elements sequencing
Foxp3	Forkhead box protein P3
FPR2	Formyl Peptide Receptor 2
GPI	Glycophosphatidylinositol
gp82	Glycoprotein 82
GDF15	Growth/Differentiation Factor 15
Gal-3	Galectin-3
GWAS	Genome-Wide Association Studies
H2B.V	Histone H2B variant V
H9c2	Rat cardiomyoblast cell line
HL-1	Atrial cardiomyocyte cell line
H3K4me	Histone H3 lysine 4 methylation
H3K9me3	Histone H3 lysine 9 trimethylation
H3K27me3	Histone H3 lysine 27 trimethylation
IFN-β	Interferon-beta
IFN-γ	Interferon-gamma
IFN-γR1	Interferon-gamma Receptor 1
IL-1β	Interleukin-1 beta
IL-6	Interleukin-6
IL-2	Interleukin-2
IL-10	Interleukin-10
IL-12	Interleukin-12
IL-17	Interleukin-17
IL-18R	Interleukin-18 Receptor
IL-22RA2	Interleukin-22 Receptor Subunit Alpha-2
IMPDH	Inosine Monophosphate Dehydrogenase
ISPA	Immune-Stimulating Particle Adjuvant
LAMP	Loop-Mediated Isothermal Amplification
MAL	MyD88-Adaptor-Like protein
MAPK	Mitogen-Activated Protein Kinase
miRNA	microRNA
MMP	Matrix Metalloproteinase
mRNA	messenger Ribonucleic Acid
MyD88	Myeloid Differentiation Primary Response 88
NF-κB	Nuclear Factor kappa-light-chain-enhancer of activated B cells
N-Lac	N-acetyllactosamine
NLRP1	NOD-Like Receptor Family Pyrin Domain-Containing 1
PARP1	Poly(ADP-ribose) Polymerase 1
PBMCs	Peripheral Blood Mononuclear Cells
piRNAs	PIWI-interacting RNAs
PTX	Pentoxifylline
PCR	Polymerase Chain Reaction
RNA	Ribonucleic Acid
RNS	Reactive Nitrogen Species
ROS	Reactive Oxygen Species
RUNX3	Runt-related transcription factor 3
SHP-1	Src Homology Region 2 Domain-Containing Phosphatase 1
Smad2	Mothers Against Decapentaplegic Homolog 2
SNPs	Single-Nucleotide Polymorphisms
STAT1	Signal Transducer and Activator of Transcription 1
STING	Stimulator of Interferon Genes
Tc24	Trypomastigote Complement 24 kDa Antigen
TcI–TcVI	*Trypanosoma cruzi* Discrete Typing Units I–VI
TcBat	Bat-associated *Trypanosoma cruzi* Discrete Typing Unit
T cells	T lymphocytes
TcIMPDH	*T. cruzi* Inosine Monophosphate Dehydrogenase
TFBSs	Transcription Factor Binding Sites
TβRI	Transforming Growth Factor beta Receptor I
TGF-β	Transforming Growth Factor beta
TGF-β1	Transforming Growth Factor beta 1
TGFBR2	Transforming Growth Factor Beta Receptor 2
TGFB	Transforming Growth Factor Beta
Th1	T helper type 1 cells
Th2	T helper type 2 cells
Th17	T helper type 17 cells
TIRAP	Toll–Interleukin 1 Receptor Domain-Containing Adaptor Protein
TLR2	Toll-Like Receptor 2
TLR4	Toll-Like Receptor 4
TLR7	Toll-Like Receptor 7
TLR9	Toll-Like Receptor 9
TNF	Tumor Necrosis Factor
TNF-α	Tumor Necrosis Factor-alpha
TNFR1	Tumor Necrosis Factor Receptor 1
TNFR2	Tumor Necrosis Factor Receptor 2
*T. cruzi*	*Trypanosoma cruzi*
tRNA	Transfer RNA
*TS*	Trans-sialidase
*TSf*	Trans-sialidase fragment
*TSA-1*	Trypomastigote Surface Antigen-1
WT	Wild-Type
